# The Conditioned Place Preference Test for Assessing Welfare Consequences and Potential Refinements in a Mouse Bladder Cancer Model

**DOI:** 10.1371/journal.pone.0103362

**Published:** 2014-08-06

**Authors:** John V. Roughan, Claire A. Coulter, Paul A. Flecknell, Huw D. Thomas, Kenneth J. Sufka

**Affiliations:** 1 Comparative Biology Centre, The Medical School, University of Newcastle, Newcastle upon Tyne, United Kingdom; 2 Northern Institute for Cancer Research, The Medical School, University of Newcastle, Newcastle upon Tyne, United Kingdom; 3 Research Institute of Pharmaceutical Sciences and Departments of Psychology and Pharmacology, Peabody Building, University of Mississippi, Oxford, Mississippi, United States of America; University of Kentucky Medical Center, United States of America

## Abstract

Most pre-clinical analgesic efficacy assays still involve nociceptive testing in rodents. This is despite concerns as to the relevance of these tests for evaluating the pain-preventative properties of drugs. More appropriate methods would target pain rather than nociception, but these are currently not available, so it remains unknown whether animal pain equates to the negatively affective and subjective/emotional state it causes in humans. Mouse cancer models are common despite the likelihood of substantial pain. We used Conditioned Place Preference (CPP) testing, assessments of thermal hyperalgesia and behaviour to determine the likelihood that MBT-2 bladder cancer impacts negatively on mouse welfare, such as by causing pain. There was no CPP to saline, but morphine preference in tumour bearing mice exceeded that seen in tumour-free controls. This occurred up to 10 days before the study end-point alongside reduced body weight, development of hyperalgesia and behaviour changes. These effects indicated mice experienced a negative welfare state caused by *malaise* (if not pain) before euthanasia. Due to the complexity of the assessments needed to demonstrate this, it is unlikely that this approach could be used for routine welfare assessment on a study-by-study basis. However, our results show mice in sufficiently similar studies are likely to benefit from more intensive severity assessment and re-evaluation of end-points with a view to implementing appropriate refinements. In this particular case, a refinement would have been to have euthanased mice at least 7 days earlier or possibly by provision of end-stage pain relief. CPP testing was found to be a helpful method to investigate the responses of mice to analgesics, possibly on a subjective level. These findings and those of other recent studies show it could be a valuable method of screening candidate analgesics for efficacy against cancer pain and possibly other pain or disease models.

## Introduction

An array of nociceptive tests are used to determine the potential efficacy of new analgesics, and laboratory rats and mice are overwhelmingly the most widely used test subjects. These tests are typically classified according to the duration and intensity of the noxious stimulus and the nature of the response. Acute or phasic nociceptive assays that apply heat or mechanical stimulation (e.g. the tail-flick and Hargreaves tests; von Frey, respectively), due to the largely reflexive nature of the responses obtained, are considered less appropriate than tonic or sub-chronic tests (e.g. formalin or carrageenan) that elicit significant and persistent inflammation [1]. However, the inadequacies of both are well known [2]. Despite the development of animal simulations of persistent pain syndromes (e.g. arthritic or neuropathic pain), concerns prevail as to their clinical relevance. These ultimately stem from a continuing lack of understanding of how animal pain equates to the human experience; being a multidimensional phenomenon including both sensory and affective emotional state changes (collectively termed ‘life quality’ changes). Theoretically at least, animal pain and analgesic assays that inform on changes in these states should afford greater translational validity. This need has driven recent calls for the development of more clinically relevant *in-vivo* models [3], [4] providing a more systems-based approach [5]. This rationale explains why affective (subjective) state assessment is increasingly being considered as a more relevant approach to establishing the welfare consequences of models where animals could be exposed to pain [6]–[8]. Cancer models are one research area where considerably more knowledge on how extensively pain impacts on welfare may be long overdue.

A large and steadily increasing number of animals (mainly mice) are used in cancer testing (471,000 in the UK in 2012; a 16% increase on the previous year [9]) with only minimal knowledge regarding potentially negative impacts on welfare. However, the guidelines that are meant to minimise suffering in these studies [10], [11] do not incorporate any evaluation of changes in underlying subjective state, and obtaining evidence of cancer pain in rodents has relied heavily on assessing underlying nociceptive changes [12]–[15], and it seems this is still the case today [16]–[19]. Our group has extensively assessed changes in naturalistic behaviours as a means of assessing post-operative pain in rodents and other species using both manual [20]–[26] and automated methods [27]–[30], or used a combination of both types of analysis [31]. However, such behaviour analysis has rarely been used to assess the effects of cancer, and those few studies that cite ‘pain behaviour’ actually mean nociceptive or nocifensive behaviours (i.e. withdrawal or guarding) rather than true spontaneous (e.g. home-cage) behaviour (see for example [14], [32]). Whichever method of data collection is used, establishing links between outcome and causation in behavioural studies is difficult because many factors can contribute to the observed changes. Changes could occur as an indirect result of procedural influences (e.g. anaesthesia, hypothermia, dehydration) or factors linked to these such as fear and associated anxiety. Nevertheless, establishing whether and when pain arises in cancer studies would have significant consequences. Firstly it would encourage a more rigorous approach to ethical decision making by enabling researchers to apply more effective cost-benefit analyses when determining if end-points are justified. Improving welfare standards in this manner would also be beneficial to studies because unalleviated pain may also jeopardise model validity. Preventing pain has been shown to be beneficial to validity in at least one cancer trial [33] and possibly other *in-vivo* research applications [34]. Cancer pain is also an area where advances in human treatments are urgently required. Pain is the most common and feared consequence of cancer and between 30–60% of oncology patients continue to report pain prior to, throughout and following treatment [35]. Indeed, it seems the outlook remains bleak despite 40 years of attempts to address the problem [36]. Clearly, cancer pain management needs to be refined, and animal cancer models provide an ideal platform for parallel progress on welfare advancement and drug discovery. Despite this, cancer models are not generally used to screen potential analgesics, with only rare exceptions [13], [37]. This may be because most cancer studies have alternative aims, but could also be due to a lack of appropriate assessment methods; i.e. those that accommodate subjective aspects.

Conditioned Place Preference (CPP) testing is widely used to evaluate the psychoactive (affective) properties of drugs in animals [38], but usually to identify abuse liability by humans. Animals are conditioned by being confined in one ‘place’ and pairing that with drug treatment, whereas in another place they are given only a placebo. As the ‘places’ have distinctly different cues (usually in colour or texture) animals progressively associate the drugs’ affect with the place where they experienced it. If this is a rewarding experience, either due to the drugs’ positively or negatively reinforcing (analgesic) effects, animals typically spend longer in, or visit the drug-paired place more frequently. Provision of analgesia is a positive experience in painful humans, so if the same applies to animals then CPP studies have potential for evaluating both the aversive properties of pain and the analgesic effects of drugs. The approach has increasingly been applied to research on pain following the first demonstration that arthritis causes enhanced CPP to morphine and the N-methyl-D-aspartate antagonist MK-801 [39]. It was subsequently shown that a bradykinin B_1_ receptor antagonist has therapeutic potential against chronic inflammatory pain [40]. A considerable time later, King et al. showed CPP is also an effective method for assessing analgesics against neuropathic pain [41]. Qu et al. illustrated the relevance of CPP to assessing subjective aspects of neuropathic pain in rats by showing that the rostral anterior cingulate cortex is essential to mediating pain affect [42]. Rutten et al. were also able to use CPP to dissociate between the positively and negatively rewarding consequences of analgesics with opioid-like affects compared to other mechanisms in a rat inflammatory (carrageenan) model [43]. Davoody et al. then showed increased CPP to clonidine (an α-2 agonist) in rats using spinal cord injury as a centralised pain model [44]. Okun et al. utilised a similar approach to show CPP to several different drugs in an osteoarthritic model [45], and most recently, Park et al. showed absence of positive reinforcement (reward) to gabapentin in normal mice, but CPP in a polyneuropathic pain model caused by antineoplastic (cisplatin) treatment [46]. Collectively, these findings clearly demonstrate that the CPP paradigm is capable of providing animals with a means to report on their awareness of pain, and so could offer a more appropriate method of evaluating its centralised (affective/subjective) as opposed to nociceptive (effective) properties.

The notion that pain could be as complex in animals (at least in mammals) as it is in humans is underlined by the fact that no single measurement criterion can (so far) effectively evaluate it. Bateson et al. originally applied a multiple measures approach to scientific problem solving (later termed ‘triangulation’) [47], citing that although “evidence obtained by different approaches was ambiguous”, “when the whole body of evidence was considered, much greater confidence could be placed on a particular meaning”. In addressing the problem of animal suffering, and accepting that “we cannot directly observe an animals’ feelings”, Bateson recommended that “we should use a variety of tasks to gain confidence in conclusions” [48]. Accordingly, studies are now increasingly applying several different measures in an attempt to characterise pain more reliably [49], [50]. In this study we adopted a comparable approach by assessing a standard welfare parameter (body weight) alongside peripheral hypersensitivity testing, monitoring spontaneous exploratory behaviour and conducting CPP testing. Despite several limitations, hyperalgesia (hypersensitivity) testing still provides a valuable secondary indicator of pain of various different modalities including cancer [12], [16], [19]. Behaviour changes are also a common response to pain, but data collection and analyses are often time consuming, especially if applied in studies that typically last several weeks; as is usually the case in cancer studies. Interpretation of these data can also be problematic because, as has already been described, there are always alternative explanations for any behavioural changes that are detected. One often cited possibility is that signs of incapacity in rodents could be hidden as an adaptive response to reduce the likelihood of predation; although to our knowledge this has never been shown unequivocally. To at least partly overcome the time limitation we used automated behaviour registration software (HomeCageScan; Clever Systems Inc., VA, USA (HCS)). This recognises a range (>20) mouse behaviours with comparable accuracy to manual analysis by a trained observer [27], [28]. In this study we contrasted findings on hyperalgesia and behaviour changes with the results of CPP testing to assess the consequences of bladder cancer on the welfare of mice; applying the concept of triangulation. So far as we are aware, no previous attempt has been made to evaluate the CPP approach as described here during tumour development; with consecutive conditioning and testing cycles. We also sought to gauge (at least hypothetically) whether this could be a useful approach to screening candidate drugs for the treatment of cancer pain in humans. We found tumour mice gained less weight, developed hyperalgesia and showed behaviour changes that were time-linked to enhanced morphine seeking in tumour-bearing mice in the 7 days preceding euthanasia. We concluded that the most likely explanation for these changes was pain. If not due to pain, at the very least the results indicated negative impacts on welfare, possibly including *malaise*. Ordinarily mice would only be removed from studies after this time, indicating a likely need for end-point refinement in this mouse model of bladder cancer. Our method of applying CPP testing could also be a potentially useful method of testing the effectiveness of analgesics intended for cancer and other conditions, especially those where pain can escalate over time.

## Methods

### Ethics Statement

All work was conducted in accordance with the Animals (Scientific Procedures) Act 1986 and was subject to local ethical review. The experiments also adhered to the guidelines of the Committee for Research and Ethical Issues of the International Association for the Study of Pain (IASP). All procedures were approved by the UK Home Office (Project Licence PPL 60/4431).

### Subjects and Husbandry

All mice were female C3H/HeN, weighed 25 to 30 g on delivery (Charles River Laboratories Inc., UK) and had free access to a commercial pelleted diet (R&M no.3 SDS LTD., Whitham, UK) and tap water in groups of 10 for one week of acclimation. They were then housed singly (Macrolon 2 cages; North Kent Plastics, UK) for another week prior to enrolment. Sawdust and wood shavings were used as bedding and cages were supplemented with ‘Sizzle Nest’, an aspen chew-block and a cardboard tube (B & K Universal). Room temperature was maintained at 21±1°C with 15–20 air changes per hour under a 12-hour light cycle (lights off at 19∶00 h). All testing was conducted between 10 am and 3 pm.

### Data Collection

The results of 3 studies using separate groups of mice are described; (1) Hargreaves (nociceptive testing) study, (2) Behaviour study and (3) Conditioned Place Preference (CPP) testing consisting of a Pilot dose ranging investigation and then a main study. The data obtained on studies 1 and 2 were from exploratory trials undertaken by colleagues of the Northern Institute for Cancer Research (NICR) using syngeneic MBT-2 (Mouse Bladder Tumour) cells derived from C3H/HeN/J mice (cells donated by Prof. Michael O’Donnell (University of Iowa, Iowa City, IA, USA) and Dr. William A. Larchian (EMH/Cleveland Clinic Prostate Centre, Cleveland, OH, USA)). The CPP data were obtained from mice specifically obtained for testing. Our colleague’s objectives were; initially to characterise the growth profile of orthotopically implanted bladder tumours (Study 1) and then to refine the implant methodology (Study 2). However, both studies were an opportunity to collect supplemental data on pathological effects (possibly including pain) that would help in the design of the main (CPP) study; thereby reducing animal use to as low a level as possible.

### Hargreaves Study

This was a pilot study undertaken to establish the growth characteristics of orthotopically implanted MBT-2 cells that would inform on the eventual design of the CPP study. Group sizes were determined by NICR colleagues based on their study requirements. Mice were randomly allocated to three groups of 5 that underwent laparotomy for orthotopic implantation of 100 µl of Dulbecco’s Phosphate Buffered Saline (DPBS), or the same volume of DPBS containing 2×10^4^ or 2×10^5^ MBT-2 cells. A fourth group of 5 mice were anaesthesia only controls. Eight mice that underwent surgery were randomly chosen to receive 5 mg/kg meloxicam (subcutaneously; s/c) to alleviate post-surgical pain [27], while the remainder received saline (0.3 mls s/c; n = 7). Anaesthesia was induced with 5% isoflurane in oxygen (5 l/min.) in a Perspex chamber. Mice then lay in dorsal recumbency on a heating blanket to maintain body temperatures between 34.5 and 37.5°C and anaesthesia was maintained with 1.5–2% isoflurane in oxygen (500 ml/min.). Depth was monitored by observing respiratory rates and regularly assessing toe-pinch reflexes. Simple eye ointment (Pliva Pharma Ltd., UK) was used to prevent corneal drying. Surgery was a 0.5 cm midline incision in the skin and muscles overlying the bladder. Once exposed, 0.1 ml of saline or the appropriate tumour cell suspension was injected intramuscularly into the bladder wall using an insulin syringe. The abdominal muscles and skin were closed separately using 5/0 polyglactin 910 (‘Vicryl’, Ethicon Ltd, Edinburgh, UK) and tissue glue (‘Nexaband’, Abbott laboratories, Chicago). Anaesthesia lasted approximately 10 minutes.

Thermal withdrawal latencies were obtained at baseline, and at 1 hour and 1 day (24 hours) following surgery, and then on days; 3, 7, 10, 14, 17, 21 and 24 using the Plantar Test (Model 37370; Ugo Basile, Italy) according to the method of Hargreaves [51]. Animals were placed in clear acrylic chambers on a glass floor and habituated for 10 minutes. An infra-red heat source was alternately applied to the mid-plantar area of each hind paw (70% of maximum heating capacity; equivalent to 253±7 mW/cm^2^; 30 second cut-off). This provided a suitable range of response latencies in normal mice (∼between 3 and 8 seconds). Three responses were obtained from each hind paw with a minimum interval of 1 minute between ipsilateral recordings. Data were only obtained from the anaesthesia control mice until day 3 when they were required elsewhere. Body weights were recorded daily until day 4, and then on nociceptive test days. As tumours were internal, growth stage was estimated by daily palpation by an experienced technician who graded tumours on a 3 point scale; from 1 (barely present) to 3 (obvious without handling). These inspections also included a record of activities associated with abdominal pain in rodents [21], [31] and other signs of ill-health such as haematuria, lethargy, dehydration and poor coat and body condition.

### Behaviour Study

The behaviour data were collected as part of a follow-up study (again without additional animal use) to assess a potentially more refined (non-surgical) method of cell implantation [52]. Our colleagues sought to establish if tumour engraftment could be improved with this method, whereas we wished to evaluate the longer term effects of cancer growth on behaviour. As before, group sizes were pre-determined; 20 mice were allocated to the DPBS group and 20 to the tumour group. Anaesthesia was induced as previously described but in batches of 5 mice using a custom-made open-mask gas delivery system. The implant procedure has already been described, but briefly, the bladder of each mouse was accessed via a 27 gauge paediatric cannula placed in the urethra. Urine was voided by gentle supra-pubic pressure and aspiration, and the bladder mucosa conditioned with a mild acid rinse (50 µl of 0.1 M HCl) for 20 seconds. This was also aspirated and then neutralised with 50 µl 0.1 M KOH, and three DPBS washes. The bladder was emptied before instilling 50 µl of 5×10^6^ MBT-2 cells or the same volume of DPBS via a syringe tied to the tail. The greater cell concentration was in anticipation of an unknown cell fraction being voided in urine following recovery. Cells were left *in-situ* for 30 minutes, following which the mice were placed in an incubator at 37°C for 20 minutes. Daily tumour development was monitored as before and body weights were also recorded daily. Mice were filmed individually for 10 minutes using a video camera (Sony DCR-HC96, Sony, Japan) positioned on a tripod 30 cm from the front of clear ‘1284’cages (35×20×14 cm; Techniplast, UK Ltd) containing only bedding (Aspen sawdust). The procedure for behaviour data collection was as previously described [28] except that it was on alternate days before tumour detection, and then daily. Filming was always during the light phase but the time of day each mouse was filmed was randomised within the tumour and control groups.

### Conditioned Place Preference Studies

**Figure 1:**
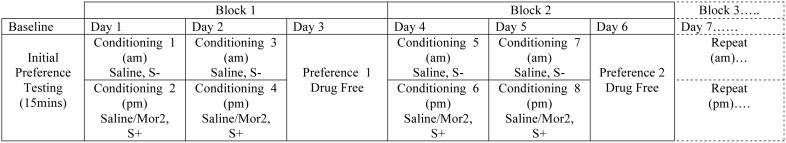
Timeline of CPP testing. Successive blocks were each of two conditioning days where mice underwent morning S− (saline) and then afternoon S+ (morphine or saline) treatments and were confined for 45 minutes in the black or white chamber. The third day of each block was a drug free preference test, and 3 day cycle was repeated until euthanasia.

The CPP work was conducted in 2 stages. The first was a pilot study to explore development of CPP to morphine under repetitive conditioning and testing cycles, and to identify a morphine dose for the main CPP study. The chosen dose would be one that supported only modest place preference but retained sufficient analgesic properties to elicit negative reinforcement in tumour bearing mice. Numbers were determined using a retrospective power analysis on previous morphine CPP results across a range of dose rates in normal mice (1 and 5 mg/kg s/c) [53] that should also elicit significant analgesic effects [54], [55]. Sample sizes of 8 or 10 were retrospectively found to achieve 79% or 91% power in detecting significant CPP to a drug (3 or 5 mg/kg morphine) versus placebo-paired compartment. The CPP apparatus consisted of black (steel rod floored) and white (steel grid floored) compartments separated by a grey (solid floored) start chamber. We used 4 identical testing units (Med Associates, St Albans, VT, USA; Model CPP-3013AT) equipped with automatically controlled guillotine doors and lights. Compartments housed infra-red arrays to automatically record compartment residence times and movements within and between the 3 compartments. An initial trial was used to determine the baseline compartment preference of each animal. For this they were placed into the central (grey) start chamber for 1 minute prior to turning on the lights and opening the guillotine doors. Preference testing was always conducted between 10 am and 2 pm. The relative time spent exploring the black and white compartments over a subsequent 15 minute trial was calculated as a proportion of the total test time (see CPP data analysis section). The mice were assigned for S+ (saline or morphine) conditioning in the compartment in which they spent proportionately the least amount of time (i.e. their non-preferred chamber), but no mice showed proportionate initial compartment preferences exceeding 0.6 (the CPP analysis section details the preference calculation method). The design was then counter-balanced such that the initial relative group preference approximated as closely as possible to 0.5 (i.e. equal numbers were assigned per group to the black (B) or white (W) S+ chambers). On this basis eight mice were allocated to each of 2 morphine groups spanning the likely range of effective analgesic dose rates (1 or 5 mg/kg s/c) and a representative group of 4 mice were saline controls. Once we reached the stage of cancer testing we knew the traditional CPP approach of repetitive conditioning trials followed by a single preference test would not be appropriate. This was because if pain occurred; we also wished to capture its onset. Informed by this, all CPP testing (including in the pilot study) consisted of repetitive 3 day blocks, with each block comprising 2 days of conditioning before a drug-free preference test each subsequent day (shown in Figure 1). For the pilot study the 4 controls received only saline (0.03 mls s/c) under both the S− and S+ conditions. Because of possible morphine ‘carry-over’ to afternoon S+ trials it was necessary that vehicle (S−) injections were always given in the morning and S+ (morphine or saline) in the afternoon. Morphine was obtained as morphine sulphate solution (30 mg/ml; NHS Supplies, UK) and was appropriately diluted with water for injection (wfi) to be administered subcutaneously in a volume of 0.03 mls. All injections were given immediately before placement into the allocated B or W compartment for a 45 minute conditioning period with the guillotine doors closed. Mice were returned to their home-cages immediately following each conditioning session. Four consecutive preference tests were undertaken for the pilot study (4 test blocks; Figure 1) precisely following the method used to determine the initial compartment preference.

The main CPP investigation began with a repeat of initial preference testing. Each mouse was assigned a B or W compartment for subsequent S−/S+ conditioning in the previously described manner (balancing numbers initially preferring the B or W compartment across the control and tumour groups). As before, mice were assigned to their initially non-preferred compartment as far as possible. Tumour mice were again implanted via the urethra with 50 µl DPBS containing 5×10^6^ MBT-2 cells. Thirty mice were orthotopically implanted with tumour cells and ten were saline controls. These numbers were informed by our previous findings on numbers of graft failures following urethral implantation. We planned to balance the design (and further conserve animal usage) by assigning any such failures to control groups, but only if there were no palpable or other signs of tumour development after 21 days. Tumour absence was also confirmed *post-mortem.* Once the clinical inspections confirmed tumour growth, each mouse began CPP conditioning and a treatment matched non-tumour control entered the study simultaneously. The 3 day series of conditioning and test sessions then continued until mice were euthanased (see section on end-point determination). Experimental groups therefore formed a 2 (Drug)×2 (Treatment) factorial design that combined conditioning to morphine or 0.9% Saline in tumour or non-tumour mice. We selected an intermediate dose of morphine (2 mg/kg s/c) for the main study based on the findings of the pilot study. In the main CPP study preference tests were also 45 instead of 15 minutes, but the methodology was otherwise identical to that used in the pilot study. Due to time constraints and the potential confound of placement into other unfamiliar environments (effectively other ‘places’), behaviour and hyperalgesia assessments were not undertaken as the CPP study progressed. However, the effect of late-stage analgesic treatment on behaviour was assessed as a potential refinement option. This meant that on euthanasia days mice were filmed for 20 minutes before and after injection of the conditioning dose of morphine (2 mg/kg s/c) and identical recordings were made from an equal number of controls on the last day of the study.

### End-point determination

Clinical inspections were conducted in all studies to determine if tumour-bearing mice needed to be withdrawn. These were in accordance with published guidelines [10], [11] and with the advice of highly experienced animal care staff. Euthanasia was unavoidable once animals had a large palpable tumour or more than 15% body weight loss, and if either of these coincided with significant haematuria (indicated by blood stained fur and/or bedding). All mice including the controls underwent palpation. They were restrained in the manner that is usual for an intraperitoneal injection. The bladder region was then gently palpated between the thumb and forefinger, but with sufficient pressure to determine the earliest time of onset of tumour development as reliably as possible.

### Data Analysis

#### Hargreaves study

As response latencies were initially similar between the left and right hind-paws, all values were averaged to calculate the overall mean response latency at each recording time. The data met the requirements for parametric analysis so were examined using repeated measures ANOVA with ‘Time’ (10 levels) and ‘Group’ (4 levels; 2×tumour; 1×placebo and anaesthesia only) as within and between-subject’s factors respectively. *Post-hoc* individual time and group comparisons were made and probability levels adjusted accordingly (*Bonferroni*). The body weight changes were also calculated as change from baseline and the same statistical methods applied. For greater clarity, the weight changes are depicted as absolute values at each time-point. Due to absence of nociceptive data in the non-surgery control mice after day 3 and body weight data after day 4 all analyses following these times included the surgery groups only. Finally, the effects of pre-surgery meloxicam were examined by comparing baseline response latencies to the 1 hour time point in the ANOVA; but with ‘pre-treatment’ (meloxicam or saline) as a between subject’s factor (n = 7 (Saline) versus n = 8 (meloxicam)).

#### Behaviour study

The behaviour data were processed using HCS automated analysis software. The measures used were total distance travelled and five behaviour classes comprising different elements of the 20 individual behaviours the system recognised. These were: 1) Rearing (comprising ‘Rear Up’ (both partial and full bipedal extension) & the reciprocal activity ‘Come Down’); 2) Active behaviour (including walking, running, jumping and climbing); 3) Abdominal Grooming, and 5) Resting (inactive periods). We summed the occurrences of behaviours within each class as the relative magnitude of class elements was sufficiently uniform. The mean frequency of each class was calculated for each mouse on each filming day. The relative duration of each class (duration/frequency) was also calculated to investigate the possibility that time spent engaging in each type of activity changed independently of its total occurrence. All behaviour data were tested and met the assumptions necessary for parametric analysis. Repeated measures ANOVA was used with ‘Time’ (Days) and ‘Group’ (Tumour versus Control) as the within and between subject’s factors for each measurement. Calculations of body weight change from baseline following non-surgical tumour implantation were evaluated again with ‘Time’ and ‘Group’ (saline or tumour) as within and between subjects factors, also using repeated measures ANOVA. Multiple comparisons between individual times were again probability corrected (*Bonferroni*).

#### Conditioned Place Preference Studies

In both the pilot study and main CPP study the initial black or white compartment preferences were calculated as the proportionate time spent in each compartment. Where t = time, the Black (B) compartment preference (Bpref) was therefore calculated as tB/(tB+tW), and the White (W) compartment preference (Wpref) as tW/(tW+tB). Thus, a proportionate score of >0.5 indicated a greater preference for that chamber and this became the S+ condition. All subsequent calculations of chamber preference were made in the same way, and the proportion of time spent exploring the S+ chamber relative to S−(tS+/((tS+)+(tS−)) was the dependent measure over successive preference tests. We again used repeated measures ANOVA to compare S+ preference scores over successive tests in the pilot and main study with ‘Time’ and ‘Group’ as respective within and between subject’s factors. The source of any individual group differences was again determined using *post-hoc* analyses and applying *Bonferroni* correction of probability levels.

## Results

All results are expressed as mean±1SEM. The electronic files pertaining to all analyses are publicly available at Dryad (http://datadryad.org/), or can be accessed by contacting the lead author.

### Hargreaves Study

**Figure 2:**
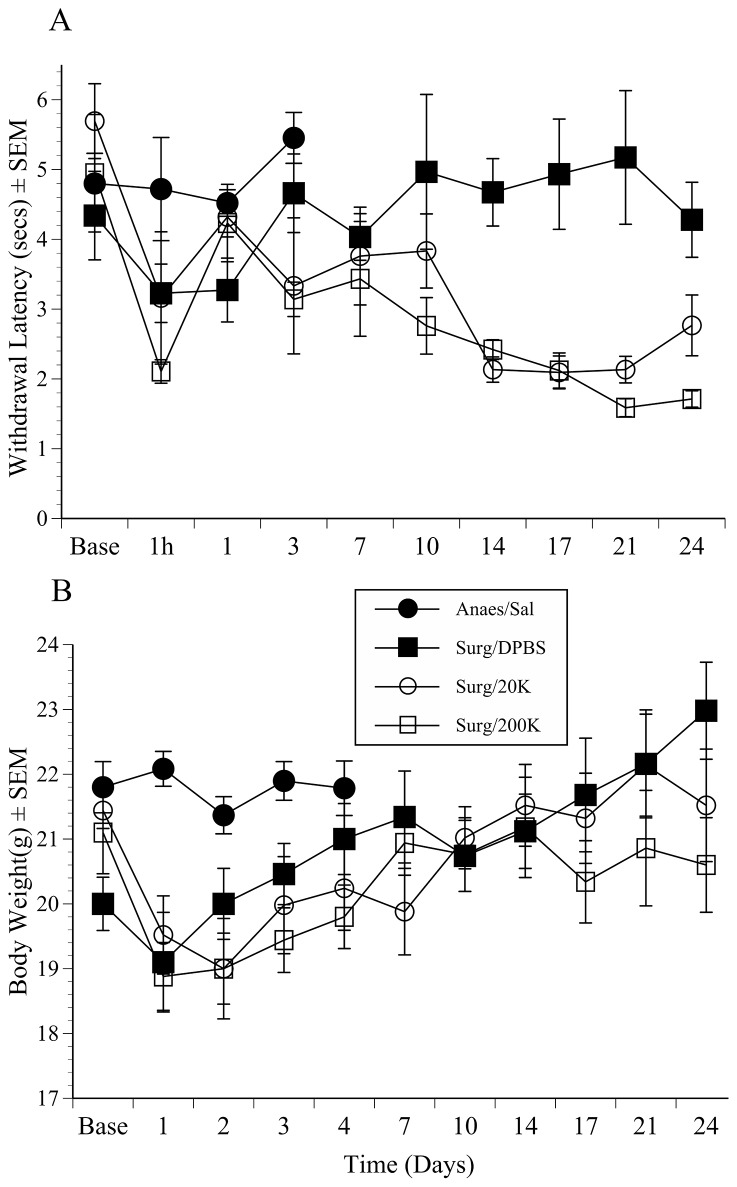
Hargreaves study results. The results of nociceptive testing (A) and recording weight changes (B) (mean±SEM) in mice that underwent anaesthesia only (Anaes), or anaesthesia followed by laparotomy for orthotopic implantation of DPBS (100 µl) or MBT-2 bladder tumour cells (2×10^4^ or 2×10^5^; 20 K or 200 K, respectively) (n = 5 per group)). (A) Hargreaves recordings were at baseline (Base), 1 hour (1 h), and then on the days indicated by the *abscissa*. Mice implanted with the highest cell concentration showed lower Hargreaves response thresholds (p = 0.02) than the anaesthesia only controls over the first 3 days (indicating hyperalgesia). Also, beginning on day 14, mice with tumours developed significant hyperalgesia compared to surgery/saline controls (p = 0.019, 0.001 (2×10^4^ or 2×10^5^ groups respectively)). (B) Tumour groups lost significantly more weight over the first 3 days than either the anaesthesia only or surgery controls (p≤0.04), but mean baseline body weight was recovered in these mice by post-operative day 4.

Three mice in the 20 K group had palpable tumours (graded 1–2) at day 14, and three in the 200 K group reached this stage by day 10. Thereafter tumour burden gradually increased, and by day 21 tumours were apparent in all animals such that in several cases tumours could be identified without need for palpation. There was no indication of a need to euthanase any mice before the pre-scheduled study end-point on day 24. As expected, the anaesthesia controls showed no adverse effects, and aside from the first day following surgery the saline/surgery controls also appeared ‘normal’. Figure 2a shows the mean change in Hargreaves withdrawal latencies (threshold to respond) in the tumour and control groups over the time course of the study. Thresholds were initially similar across the 4 groups; between 4.5 and 5.5 seconds; indicating equivalent nociceptive status initially. Although withdrawal occurred significantly earlier at the 1 hour time-point in the surgery groups (5±0.4 vs 3±0.3 seconds; (F(1,13) = 16.6, p = 0.001), by 24 hours these showed thresholds that were no longer significantly different from baseline. This was also the case in in the anaesthesia controls over their 3 post-treatment recording days. The surgery controls had also apparently recovered by day 3, and over the time course of the study their response thresholds remained relatively consistent; from 4.3±1.4 at baseline compared to 4.3±1.2 s on day 24. By contrast, mice implanted with tumours showed an overall increase in thermal sensitivity (thresholds/latency to respond reduced). These effects led to a significant overall ‘|Group’ effect (Tumour vs Control; (F(1,12) = 12.2, p = 0.001) and ‘Group x Time’ interaction (F(18,108) = 2.6, p = 0.001). *Post hoc* analyses showed both tumour groups had significantly reduced thresholds compared to DPBS controls arising on day 14 (p = 0.019, 0.001, 20 K and 200 K vs. DPBS, respectively). This was maintained until day 21 in both groups, but by day 24 only the 200 K group showed significantly reduced response latencies relative to controls (p = 0.002). There were no significant effects of meloxicam (positive or otherwise) on either withdrawal thresholds or body weight (data therefore not shown).

As shown in Figure 2b Surgery/Saline mice were somewhat lighter initially, but not significantly so (∼1.5 g). To account for this ANOVA with individual (*post-hoc*) comparisons was conducted on calculations of weight change from baseline over the first 3 days. Post-surgery losses averaged 1.7±0.3 g overall (F(1,13) = 32.4, p<0.001). Weights were maintained in the non-surgery mice. Both tumour (surgery) groups showed this weight loss from 1 to 3 days, with the greatest mean loss being 2.2±0.8 g in the 200 K tumour group on day 1. The 20 K and 200 K tumour groups both lost significantly more weight than the anaesthesia controls on post-surgical days 1 and 2 (p = 0.01, 0.003 (day 1); 0.01, 0.04 (day 2), 20, 200 K groups respectively). The same comparison between the surgery and anaesthesia controls was not significant, probably because the surgery controls were initially lighter. The surgery controls therefore lost seemingly less weight immediately, and had recovered more weight by day 3 than either the 20 K (p = 0.038) or 200 K tumour groups (p = 0.018). By day 4 there were no significant group differences. The pattern was then of increased weight until the end of the study. This resulted in a significant effect of ‘Time’ (F(6,72) = 7.1, p = 0.002). Groups showed only a modest (∼1.5–2 g) increase in weight, although by the end of the study (days 17 to 24) there was evidence that weight gain slowed more in the high cell density group than the other surgery groups. This greater overall reduction in weight in the last week in the high density (200 K) tumour group, and over the last 3 days in the 20 K group resulted in a significant ‘Group×Time’ interaction (F(2,12) = 4, p = 0.045).

### Behaviour Study

**Figure 3:**
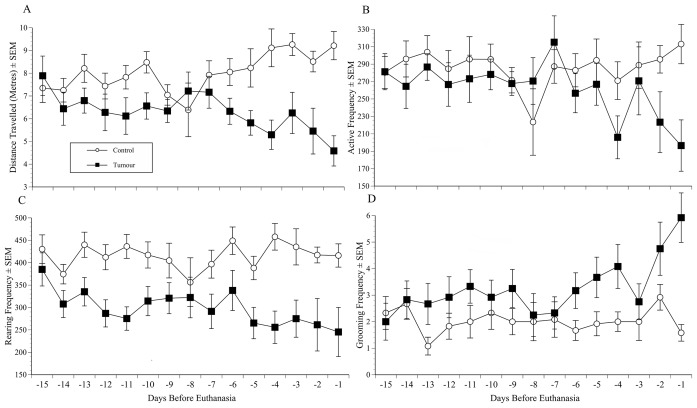
Behaviour study results – Changes preceding euthanasia. Results of automated behaviour analysis (HCS) during the period 15 to 1 day prior to euthanasia in the Hargreaves study (mean±SEM). (A) Tumour mice travelled significantly less distance overall (‘Group’, p = 0.017) and showed a pronounced decline over the 8 days before euthanasia (‘Group×Time’, p = 0.001). (B) Active behaviour showed a decline during the days preceding euthanasia (−3 to −1). (C) Tumour mice reared less over the entire 2 weeks prior to euthanasia than controls (‘Group’, p = 0.001) and (D) groomed more overall (‘Group’, p = 0.038), but more intensively so over the final 7 study days (‘Group×Time’, p = 0.048).

Of the 20 mice implanted for the behaviour study (non-surgically) 12 (60%) developed tumours. The implant procedure caused only marginal weight loss (0.8±0.1 g), and all mice fully recovered their starting weights by day 2. The time to detect a solid tumour mass was relatively uniform (28±1.7 days), but subsequent tumour development was considerably more varied. The time from tumour detection to reaching end-point criteria ranged from 10 to 23 days (mean 13.6±3.5). The first mouse was euthanased on day 37 following implantation and 2 mice survived without obvious concerns for 50 days (when the study ended). An inverse relationship was predicted between *ex-vivo* tumour wet weights and survival time (days from detection to euthanasia), but no such relationship was found; average tumour mass was 1.1±0.17 g, range 0.4 to 2 g. The net effect of the varied tumour growth rate was that numbers in the tumour groups were progressively depleted, so aside from immediately following implantation, plotting results on a conventional ascending timescale was not helpful. For both the behaviour and body weight data we therefore examined the results in reverse of the time (day) mice were euthanased; predicting this would be the time when the tumour and control groups would be most distinct. The data were first standardised by matching those from the tumour group to equivalent control data (in terms of elapsed study time). Figure 3 shows the behaviour results from the last 15 days before each mouse was euthanased, with data from an equivalent elapsed study time in controls mice. Panels 3a–d respectively show distance travelled, active, rearing and grooming behaviour frequency. The ANOVA showed tumour bearing mice travelled significantly less overall (Figure 3a; F(1,22) = 6.65, p = 0.017), and the group difference widened as time progressed. This was especially obvious from 7 days prior to euthanasia when the tumour mice began a marked decline in activity, whereas the opposite effect was observed in controls, leading to a significant ‘Group×Time’ interaction (F(14, 308) = 4.1, p = 0.001). There were less obvious group differences in active behaviour (Figure 3b). The groups were not significantly different overall, but the pattern of increasing activity in controls versus a decline in tumour mice was maintained, but was only marginally significant (‘Group×Time’; F(14,308) = 2.3, p = 0.04). The last 7 days was again the time when group differences were most apparent, however, there was a noticeable spike in active behaviour in tumour mice 3 days prior to euthanasia that partially impacted on the distance measure. Rearing behaviour showed the most obvious group separation, with the tumour mice rearing less during the entire 2 weeks prior to euthanasia (Figure 3c; F(1,22) = 14.3, p = 0.001). There was no particularly obvious decline in tumour versus control mice over the terminal 7 days, but rearing was maintained at a markedly greater frequency in controls (416±90 vs. 245±189). Grooming showed a slight overall increase in both groups over time (‘Time’ factor significant; F(14,308) = 2.1, p = 0.05; Figure 3d). The tumour mice also groomed more frequently overall (F1,22) = 4.9, p = 0.038), but again, the major effect in tumour mice was an increase in grooming frequency over the final 7 days (significant ‘Group x Time’ interaction; F(14,308) = 2.1, p = 0.048). This result was rendered only modestly significant due to another incongruous effect 3 days prior to euthanasia when grooming frequency fell in tumour mice. Rest periods (inactive behaviour) were no more frequent in control or tumour mice until the final 3 days when tumour mice tended to show a reduction compared to only a very slight increase in controls (not shown).

**Figure 4:**
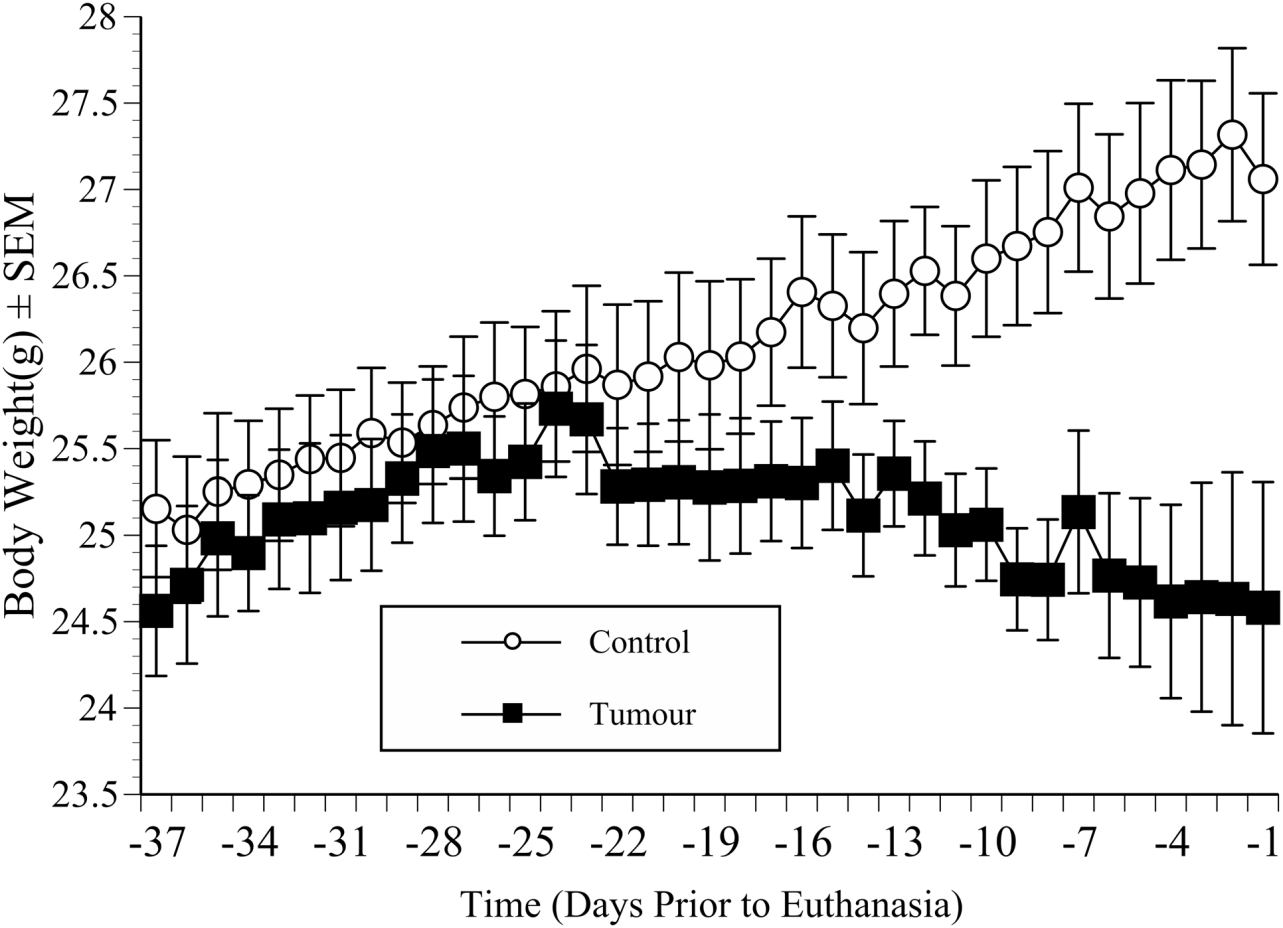
Body weight in catheterised mice (implanted non-surgically). The mean (±SEM) body weight of groups of mice implanted orthotopically with either MBT-2 tumours (5×10^6 ^cells/mouse; 50 µl) or an equal volume of saline, and used to collect behaviour data (n = 12 per group; graft successes only). As in Figure 3, data on days prior to euthanasia are shown following time matching (equivalent elapsed study time). Tumour-bearing mice were significantly lighter than controls from day 12 (‘Group’; p = 0.008) and progressively lost more weight until the study end.

The body weight data on the day each mouse was euthanased were matched as previously described: with results in tumour mice matched to data from an equivalent elapsed study time in a randomly chosen non-tumour control. This was repeated for each day prior to euthanasia. Figure 4 shows the mean body weights of the control and tumour groups from 37 days prior to euthanasia. The pre-study weights of the 2 groups were not significantly different. Both groups were gaining weight 37 days prior to the eventual study end, however, the tumour group stopped gaining weight 2 weeks later (22 days before euthanasia). ANOVA showed a significant ‘Group×Time’ interaction 12 days prior to euthanasia (F(1,23) = 8.55, p = 0.008). *Post-hoc* analyses (independent samples t-tests) showed that by this time the mean weight of tumour bearing mice was 25±1.1 g, compared to 26±1.3 g in controls (p = 0.015). The control mice then steadily increased in weight whereas the reverse occurred in the tumour group (or at least they continued to fail to gain weight). By the end of the study (on day −1) the mean weight of control mice was 27±1.8 g compared to 24.5±2.5 g in the tumour group (p = 0.009). In the tumour mice this amounted to a proportional weight loss of 4.6% compared to the time when they were heaviest (Day 24 prior to euthanasia). We tried to minimise any masking effect of underlying tumour burden on this weight estimate by subtracting *ex-vivo* tumour wet weights, but did not find mice with larger tumours lost significantly more weight.

### Conditioned Place Preference Studies

**Figure 5:**
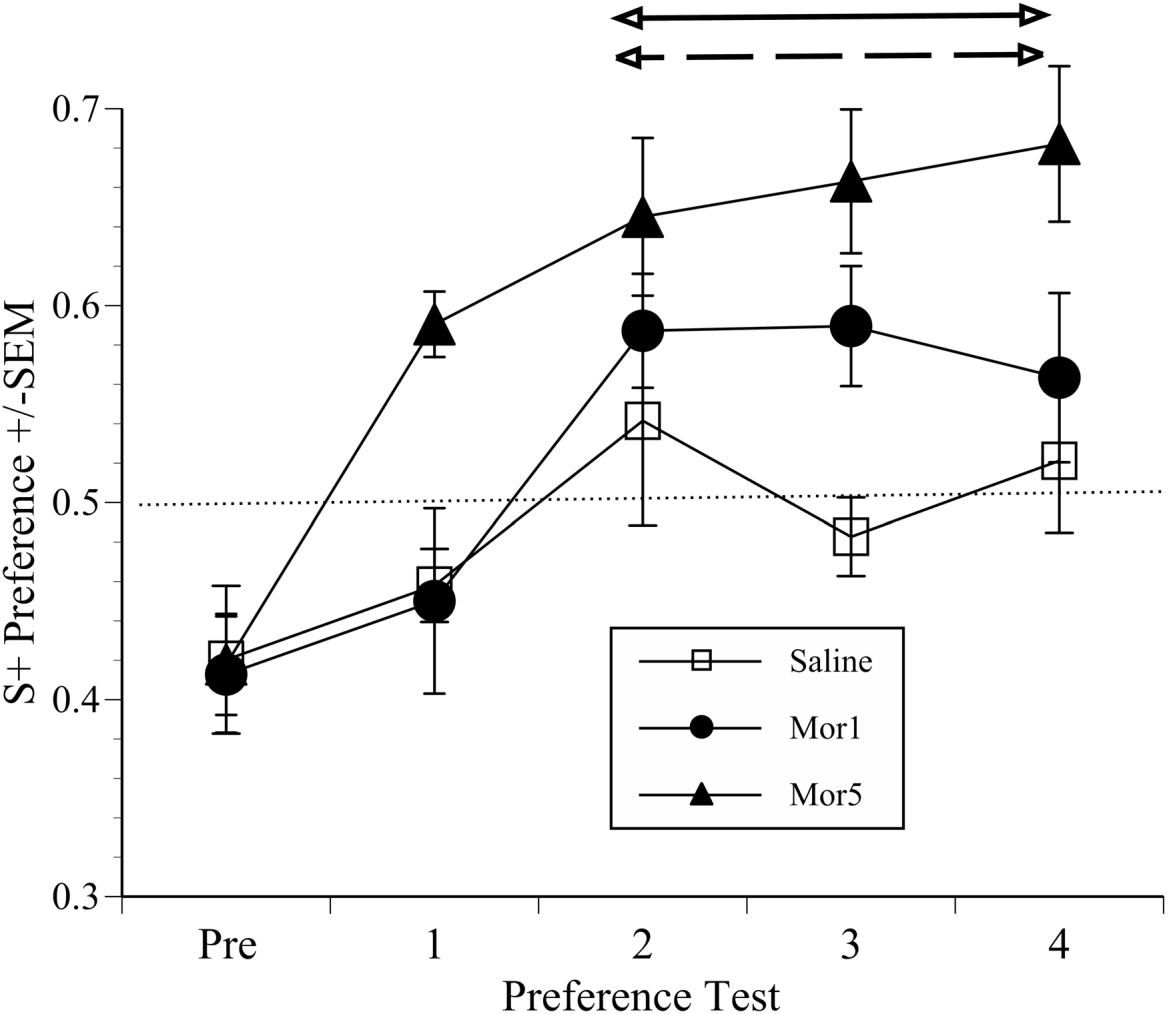
CPP pilot study results. Mean S+ chamber preference scores (±SEM) across 4 successive tests in mice conditioned to saline (0.03 mls; n = 4) or morphine (Mor) at 1 or 5 mg/kg (n = 8/group). Mice were conditioned in their initially non-preferred chamber (Pre). Scores above 0.5 (dotted line) indicate enhanced S+ (morphine or saline) chamber preference. Arrowed lines at the top of the chart denote the time period over which the preference for 5 mg/kg morphine exceeded 1 mg/kg (p<0.05; dashed arrowed line) or saline (p<0.01; solid arrowed line).

The results of the CPP pilot study are shown in Figure 5 according to mean S+ preference scores of the groups given saline and each morphine dose. ANOVA indicated a significant overall ‘Group’ difference (F(1,2) = 5.6, p = 0.013). The *post-hoc* analysis showed mice given 5 mg/kg morphine had significantly elevated S+ scores over tests 2 to 4 compared to the saline controls (p = 0.005) or the mice given the lower morphine dose (p = 0.041). Saline was predictably ineffective, but as 1 mg/kg morphine was also not different from saline an intermediate morphine dose of 2 mg/kg was chosen for the main CPP study.

Implant success in the main CPP study was 70% with 21 of the 30 implanted mice developing tumours. As planned, four non-growth mice were randomly assigned to control groups, one of which subsequently developed a tumour (so data were rejected). Although overall engraftment was improved from that seen in the behaviour study, the time from implantation to detection of a solid tumour mass over the bladder was more varied. Of the mice where grafts were successful, 75% presented tumours within 14–21 days. Subsequent rates of tumour development were also varied, leading to a broad range of times from tumour detection to the time that mice were euthanased (13–46 days). This meant the number of tests conducted in tumour mice before they reached the endpoint criteria ranged from only 4 to 15 CPP trials (mean ± SD; 8.4±2.73). To minimise the impact of this variation we re-applied the selection procedure to focus on the times likely to reveal group differences; where tumour-bearing mice should show an enhanced morphine preference if they were experiencing pain. As the minimum number of tests was four, the results of the last 4 tests prior to euthanasia were examined (i.e. CPP tests −4, −3, −2 & −1); equivalent to days 10, 7, 4 and 1 day before the study ended. As in the behaviour analysis, data from the non-tumour control (saline or morphine) groups were time-matched as far as was possible to the total number of cycles of conditioning/preference testing undergone by individuals in the equivalent tumour groups.

**Figure 6:**
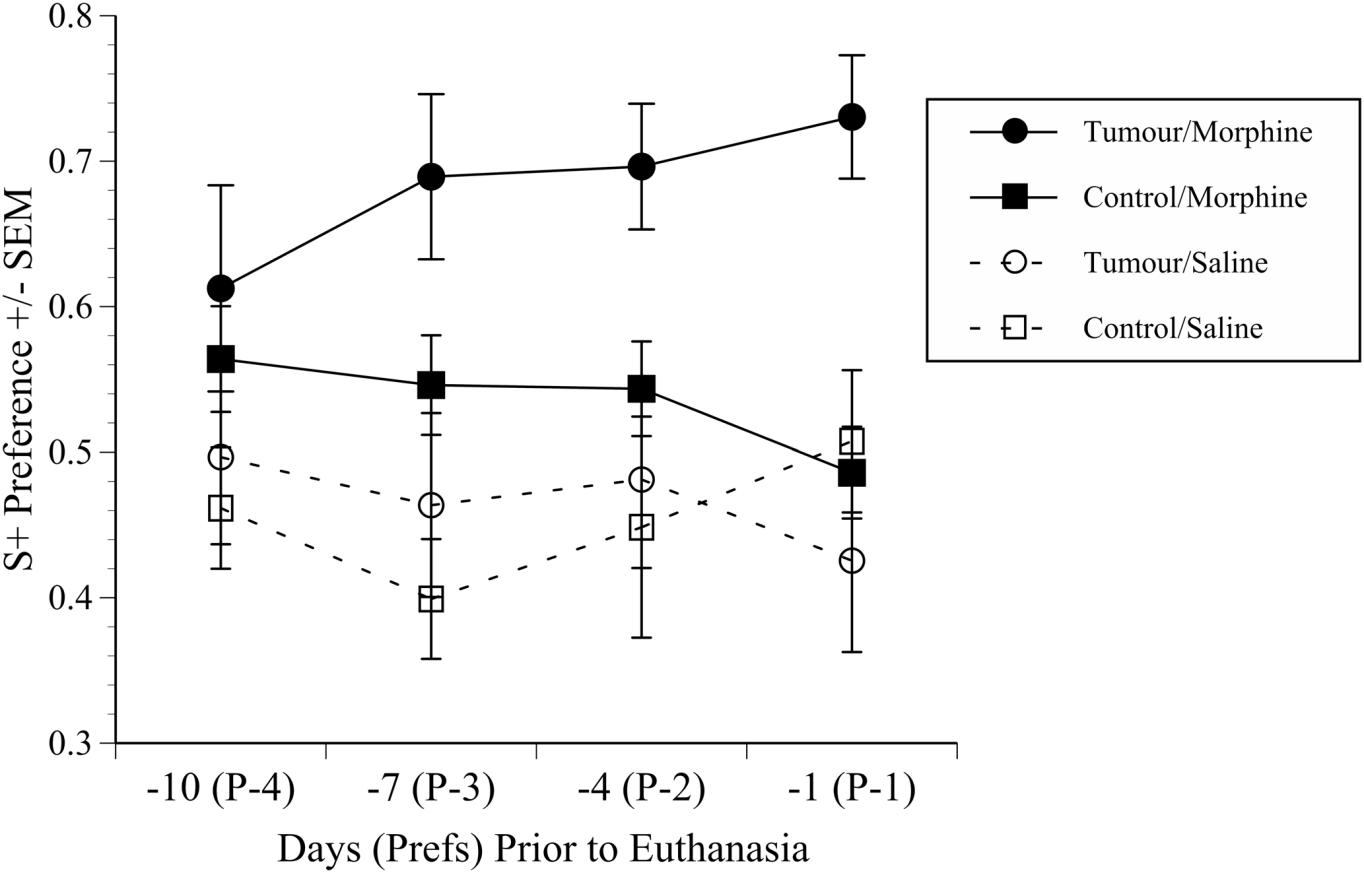
CPP morphine preference scores preceding euthanasia. Mean S+ chamber preference scores (±SEM) during the last 4 preference tests (actual days in brackets) prior to euthanasia (Day −1(1)) in normal (Control) and tumour bearing (Cancer) groups conditioned to Saline or Morphine (2 mg/kg; s/c). Scores above 0.5 indicate an enhanced S+ chamber preference. Note especially the cancer mice conditioned to morphine show enhanced morphine seeking (higher CPP S+ score) than either the saline or tumour control groups (p = 0.005; p<0.001, respectively), but from days −7 to −1 prior to euthanasia their morphine chamber preference also significantly exceeded the morphine preference of controls conditioned to morphine (p = 0.02).

There were no significant differences in S+ scores between the morphine and saline treated mice on the 3 test days after tumour detection, and both the normal and tumour bearing mice trained only to saline displayed essentially equal preference to the S+ and S− chambers over these trials (i.e. preference scores close to 0.5; data not shown). This illustrated that the apparatus and study design were unbiased. As predicted, the responses to morphine were clearer over the last 4 preference trials (Figure 6). From 10 to 4 days before euthanasia (preference tests −4 to −1) there was little change in morphine preference in controls, but the morphine-treated tumour mice displayed the highest S+ place preference scores of all four groups; the combined effect being an overall significant effect of drug treatment (F(1,29) = 4.9, p = 0.035). The morphine preference in the tumour group increased over the final 3 preference tests (7 days). Mean S+ preference over this time was calculated and results compared between groups using ANOVA. This also showed a significant overall treatment effect (F(3,29) = 6.74, p = 0.001). *Post-hoc* tests showed tumour mice conditioned to morphine had significantly elevated S+ scores in comparison to both the saline controls (p = 0.005) and the saline tumour mice (p<0.001). The key comparison, however, was between the morphine preference scores of the tumour and control mice. Mice with tumours had a significantly greater preference for the morphine paired chamber than the morphine controls (p = 0.02). These findings were consistent with the notion that mice in pain will show a place preference to a compartment paired with an analgesic drug over one paired with only saline, but crucially, more so than the drug preference shown by normal mice.

**Figure 7:**
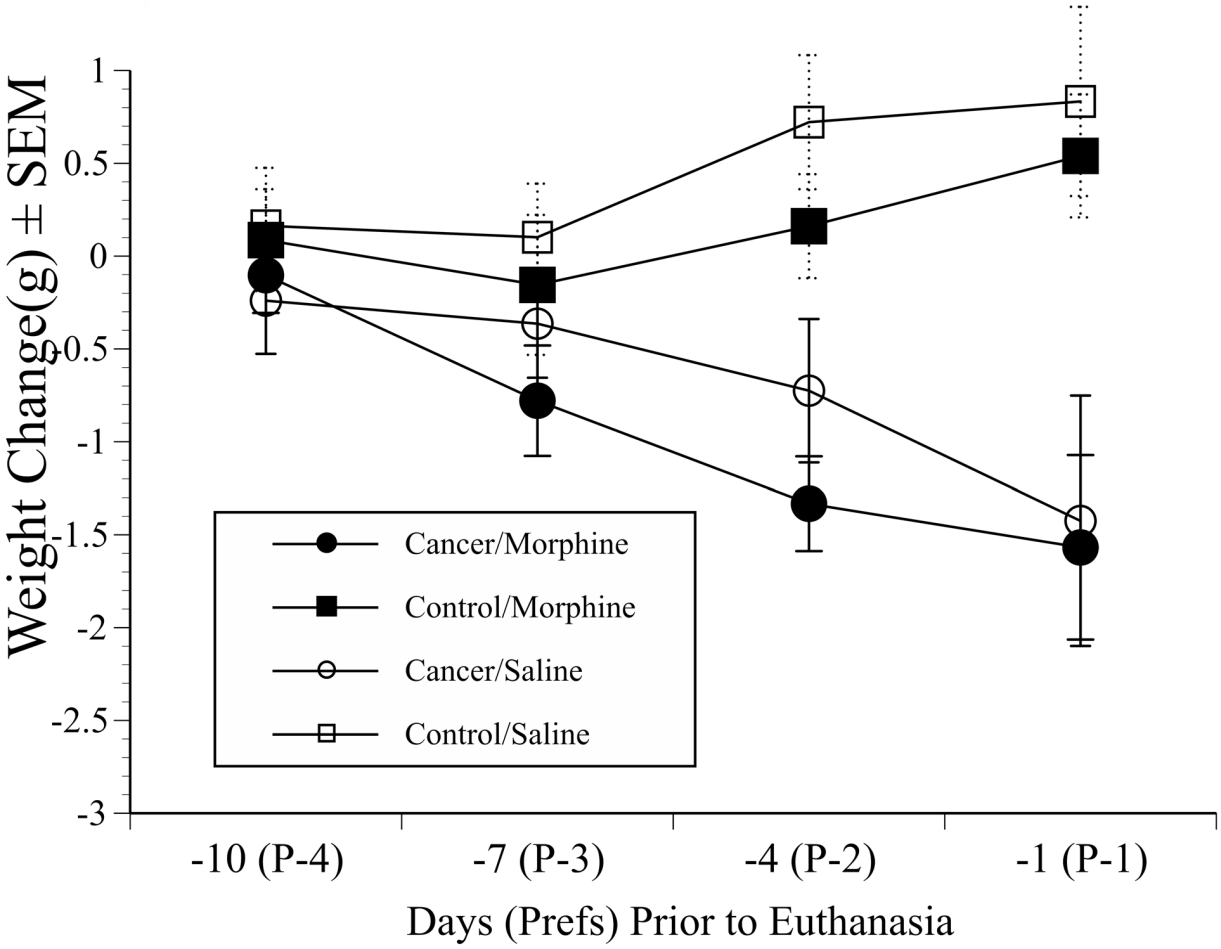
Body weight change in mice used in CPP testing. Mean weight (±SEM) on days −10 to −1 prior to euthanasia relative to weight observed on day −14 in mice in the main CPP study. Weights in tumour-bearing mice significantly declined over the final 7 study days compared to control groups (‘Group (Cancer vs Control)×Time’, p = 0.001), with no apparent effect of morphine.

Analysis of the body weight data indicated there were no significant group differences at baseline (before cell implantation). Body weights from the time period over which the CPP results indicated was the most important phase of the study were then assessed; the final 4 preferences tests (Figure 7). We used calculations of change in weight from before the previous test day (14 days prior to euthanasia). Repeated measures ANOVA was used with ‘Time’ (4 levels) as a within-subjects factor, and both ‘Drug’ (morphine or saline) and ‘Treatment’ (cancer or control) as between subject’s factors. Over the final 10 days of the study the tumour bearing mice showed a small but nevertheless significant reduction in weight (1.6±0.8) compared to non-cancer mice. The non-cancer groups either maintained body weight or showed a marginal weight increase during this period (0.6±1 g) (significant ‘Time×Treatment’ interaction; F(2,50) = 11, p<0.001). Morphine had no significant effects on body weight.

**Figure 8:**
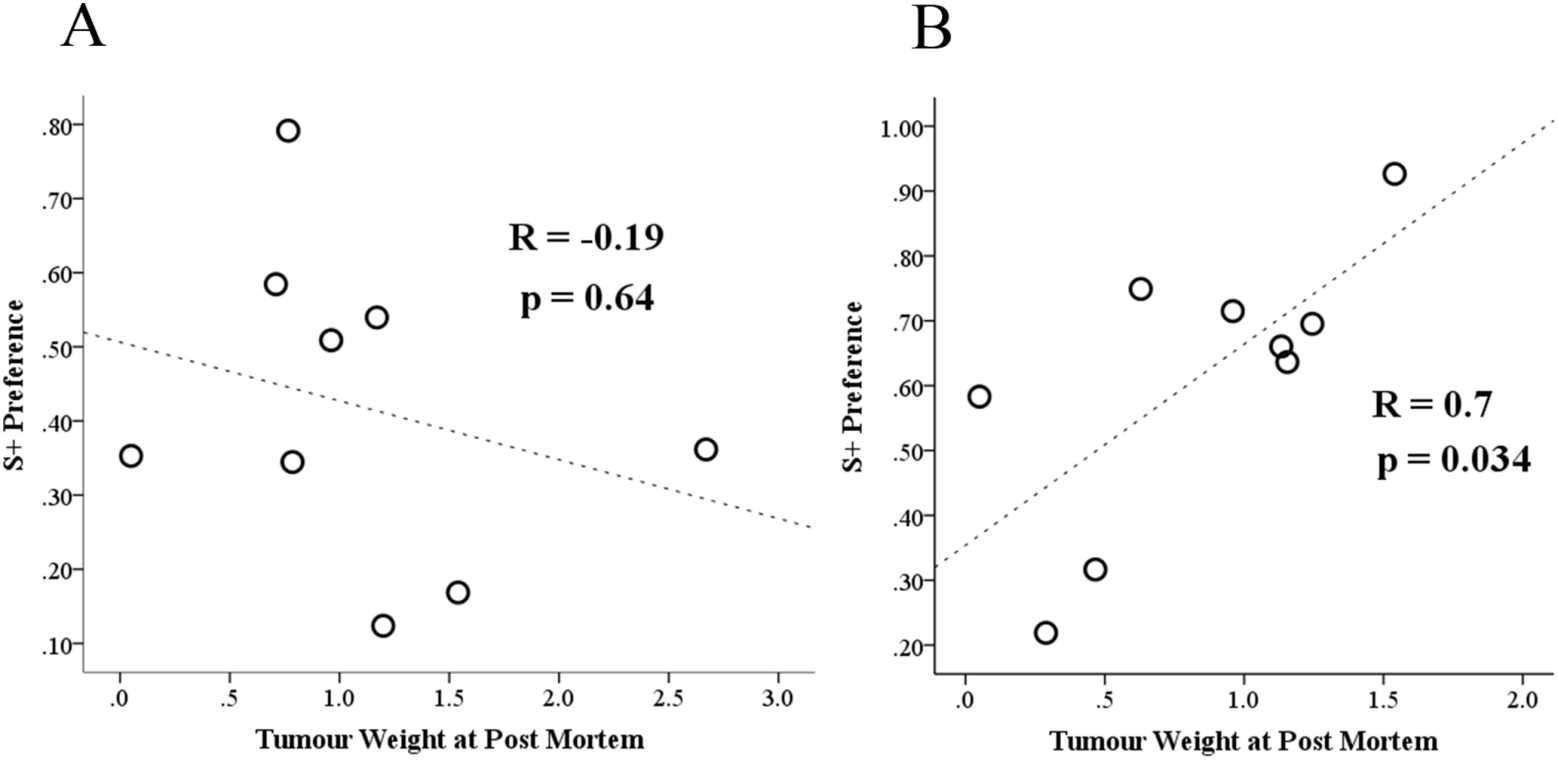
Relationship between morphine seeking (CPP score) and tumour burden. Scatterplots showing: (A) the relationship between relative S+ preference scores for saline on the day immediately prior to euthanasia versus post-mortem (wet) tumour weights (day P-1; Pearson’s Correlation); and (B) the same relationship between tumour weight and S+ chamber preference on day P-1 for mice exposed to morphine. Tumour mass bore no significant relationship with saline chamber preference, but as panel B shows, morphine seeking significantly increased with increasing tumour burden.

There was only a marginal difference in mean tumour burden between the saline and morphine groups; tumour wet-weight at *post-mortem* was 1.04±0.75 in the saline group versus 0.83±0.45 g in mice conditioned to morphine. An independent samples t-test showed morphine was unlikely to be responsible for this very slight difference (t(16) = 0.91, p = 0.4). We initially explored whether tumour burden was a significant contributor to morphine chamber preference on the last CPP test (Preference test P-1). Pearson’s correlation showed no significant relationship between S+ preference scores and tumour burden in the saline group (r = −0.19; p = 0.64; Figure 8a), however, tumour burden was significantly positively correlated with preference for the morphine paired chamber (r = 0.70; p = 0.034; Figure 8b). The same relationship between tumour burden and morphine seeking was also apparent at 4 days (Preference test P-2) before euthanasia (r = 0.72; p = 0.029 (not shown)). However, the same comparison at the P-2 time point in mice conditioned to saline was not significant (r = 0.22; p = 0.59) (P-2 data not drawn)). These results therefore showed that increasing tumour burden made a positive contribution to morphine chamber preference.

**Figure 9:**
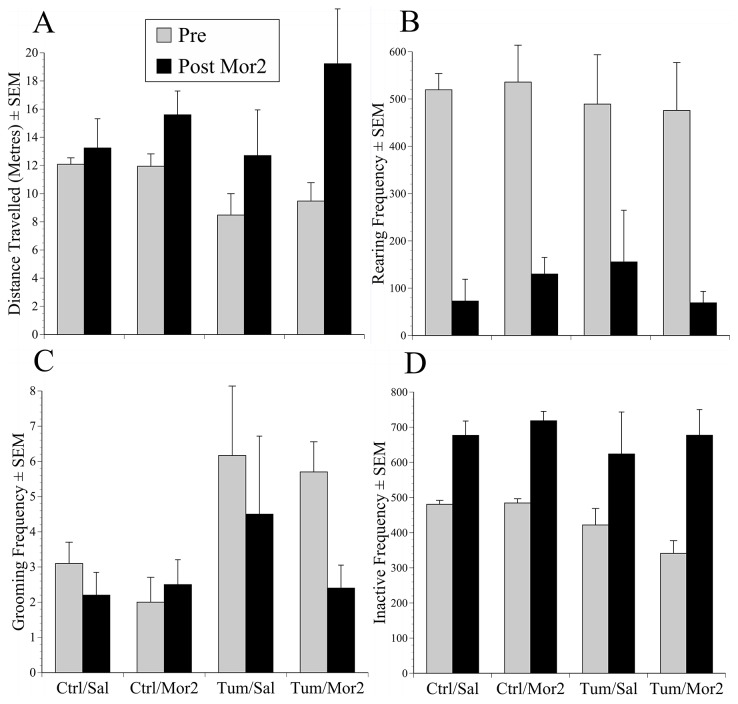
Behaviour changes following terminal morphine. Behaviour results showing the mean (±SEM) frequency before and 30 minutes after terminal-stage treatment with morphine (2 mg/kg s/c) in tumour (Tum) or control (Ctrl) mice that underwent CPP testing and were conditioned to morphine (Mor2) or saline (Sal). (A) Distance travelled; (B) Rearing; (C) Grooming; (D) Inactive periods (Pre) and following (Post) morphine treatment (2 mg/kg s/c). Morphine significantly increased overall activity, indicated by increased distance travelled and reduced rearing (‘Time’; p = 0.005; p = 0.001, Distance and Rearing respectively). Grooming was reduced by morphine but not significantly, and inactive frequency increased (‘Time’, p<0.001, respectively).

The final phase of data collection in the CPP study was to determine whether we could recognise any behaviour-based evidence of reduced pain following terminal morphine treatment. We assessed the effect of the training dose of morphine given on the last study day on grooming, rearing and inactive behaviour frequency (Figure 9). For reasons previously given, total distance travelled was the only measure needed to evaluate changes in active behaviour. The analysis used ‘Time’ (pre and post-morphine) and ‘Group’ (tumour vs. non-tumour) as within and between subject’s factors, and *post-hoc* analyses (Bonferroni) identified individual group differences. Prior to morphine the tumour-bearing mice travelled significantly less distance than controls (F(1.28) = 6.4, p = 0.017; Figure 9a). This increased in all mice following morphine (‘Time’ factor significant; F(1, 28) = 9.2, p = 0.005). Rearing frequency was similar across all groups initially, and all groups showed significantly reduced rearing behaviour following morphine (‘Time’ factor significant; F(1,27) = 83, p<0.001; Figure 9b). The mice with tumours initially groomed more than controls (F(1,28) = 104, p = 0.001), and morphine had no significant effect on this in either control group, and no significantly greater effect in tumour mice irrespective of whether they had previously been exposed to morphine (Figure 9c). Inactive behaviour was significantly more frequent in the controls versus the tumour groups prior to morphine (F(1,28) = 11.2, p = 0.002; Figure 9d), but all mice rested more frequently following drug treatment (F(1,28) = 36.7, p<0.001).

## Discussion

The work had two main aims. The first was to determine whether a relatively common mouse bladder cancer model had negative impacts on welfare, including effects caused by *malaise*, anxiety and ultimately pain. If there were any such effects we hoped to highlight these and provide information on potential refinements to prevent possible suffering in similar studies in the future. A second aim was to determine whether the Conditioned Place Preference (CPP) test provides a potentially more relevant alternative to testing analgesics against cancer pain than other presently available methods. The work consisted of a series of studies where in addition to CPP testing, we investigated the development of hyperalgesia, behaviour changes and potential prophylactic effects of morphine in terminal-stage cancer-bearing mice.

In the first study the Hargreaves method was used to evaluate any development of thermal hyperalgesia over a 24 day period following surgical implantation of bladder tumour cells. We found withdrawal thresholds reduced between 10 to 14 days prior to euthanasia, and this hyperalgesic effect was maintained until the end of the study. This increase in peripheral sensitisation provided our first evidence that this type of cancer might progressively impact negatively on welfare. A subsequent investigation examined changes in the behaviour of mice implanted with tumours via a potentially more refined non-surgical approach. Recordings of behaviour were processed using an automated system. This showed abnormal behaviour changes began between 7 and 10 days before the study ended, with the most notable effects being reduced distance travelled, reduced active and rearing behaviour and increased abdominal grooming. Changes to behaviours such as these have previously been attributed to abdominal pain in mice [30], [31], [56] and are similar to those that occur post-operatively in rats [21]. However, the most conclusive evidence that the mice actually experienced pain came from the CPP study. Morphine seeking was found to be significantly positively linked to tumour burden, but most importantly, tumour bearing mice developed a greater preference for the morphine-paired chamber than any other group. They may also have found the placebo-paired chamber aversive, but the pattern of drug seeking strongly indicated the change in compartment preference was due to the negatively reinforcing (pain relieving) properties of morphine. As with the nociceptive and behaviour changes, the changes in morphine-seeking primarily emerged during the last 10 days of the study (4 preference tests). This not only indicated the presence of pain, but showed CPP testing could be a potentially effective and relevant means of evaluating analgesics against cancer and other types of pain.

Our findings were obtained despite a number of challenges, mainly the variable success of implanting tumours and the subsequent rates of cancer growth. Although engraftment was 100% using the surgical approach, it dropped to only 60% on our first attempt to implant via the urethra, then improved to 70% in the CPP study. There were several reasons why we persisted with non-surgical implantation of tumours despite the lower graft success. This method initially offered the possibility of being less costly, both financially and in terms of welfare. Conditioning of the bladder epithelium using a mild acid rinse and subsequent buffering was meant to maintain graft success but be less invasive. Non-surgical implantation also meant there was a reduced likelihood of needing to provide peri-operative analgesics; an issue that often concerns researchers who wish to rule out non-specific influences on tumour development. Potential welfare benefits were indicated by an absence of post-procedural weight loss compared to the effects of surgery. Mice that underwent surgical implantation also showed reduced thermal response thresholds, both immediately following surgery and during the latter stage of cancer growth; albeit unfortunate that we had no comparable nociceptive data from the catheterised mice. Finally, stress such as caused by surgery is a known tumour growth promoter [33], [57], and any more rapid development could have been detrimental to the eventual timescale for CPP testing. Although tumour seeding was lower and subsequent growth slower via the urethral route, a positive aspect was that this allowed more time for CPP conditioning and also accommodated our preferred approach of only recruiting mice once tumours were detected. Establishing the most appropriate time to begin CPP testing was important since conditioning disease/pain-free mice to morphine could have had other unwanted consequences; e.g. tolerance issues or opioid-induced hyperalgesia. Avoiding these may have been important in our eventual ability to illustrate greater morphine seeking in tumour mice. Directly incorporating nociceptive testing was initially considered in determining when to begin conditioning (i.e. when thresholds reduced), but would have added a further unknown and potentially confounding variable; effectively a third compartment/place to which mice would have been exposed for relatively long periods. From an oncology perspective, however, implanting mice via the urethra may not have been the best option. This is because there are also valid welfare arguments in favour of surgical implantation, especially when tumour growth is more varied. Such variation could increase the number of animals needed to achieve adequate statistical power; making it even more challenging to determine an appropriate study end-point. The longer study duration would also increase facility (housing/husbandry) costs; an obviously important factor in overall cost-benefit analyses.

The behaviour study was meant to establish if bladder cancer impacted on aspects of behaviour in a similar way to other sources of abdominal pain in mice (e.g. vasectomy [31]), and thus required extensive collection of video footage for automated analysis using HCS software. We have previously used this system to assess the behaviour of mice, and shown it can provide precision equivalent to manual scoring [27], [28]. However, this was where the varied tumour growth had its first major impact; forcing us to apply the data matching procedure to meaningfully analyse the results. We evaluated responses across 5 behaviour categories during the 2 weeks before euthanasia. This was the period of tumour growth considered most likely to encompass the time of development of any tumour-related pain. Abdominal grooming was more frequent in tumour-bearing mice, whereas distance travelled was reduced, and active behaviour and rearing all occurred substantially less frequently than in controls, especially over the final 7 days prior to euthanasia (Figure 3). There were, however, some unexpected findings. There was an increase in active behaviour and reduced grooming in tumour mice 3 days before euthanasia (Figures 3b and d), and another decline in active behaviour in controls on day −8. We looked for some unplanned event as a possible explanation (e.g. unscheduled husbandry), but the laboratory records held no clues. An adverse reaction to palpation was also unlikely as this was repeated on each of the 2 days that followed without any apparent impact. The effects were also unlikely to have been due to the data manipulation procedure as the period over which matching was conducted spanned 2 weeks and 7 separate dates. Indeed, we cannot offer a more rational explanation, and can only state that some mice were prone to some unusual activity patterns. Nevertheless, the clear disparity between the control and tumour mice over the period preceding euthanasia highlighted this as a key period for later evaluation in the CPP study.

The morphine doses chosen for the CPP pilot study (1 and 5 mg/kg) was another key decision. This was based on evidence of significant anti-nociceptive effects in both phasic (thermal) and tonic (abdominal writhing) tests in mice [54], [55]. The larger dose had also previously been shown to elicit relatively robust CPP in rats, whereas 1 mg/kg was only marginally effective [39]. As non-tumour mice in the pilot study developed CPP to 5.0 or 1.0 mg/kg morphine in as a few as two to four conditioning trials (Figure 5) an intermediate dose of 2 mg/kg was chosen for the main study. However, the apparent speed of development of CPP, even in mice given 1 mg/kg, necessitated extending the amount of time used for later preference trials. Exploration time was increased to 45 minutes in the main study, however, only the first 15 minutes served as the dependent measure of preference. This was done in an attempt to minimise CPP development in non-tumour control mice as drug-free tests of this length can serve to facilitate extinction. The design of the main CPP experiment was informed by the original studies where the potential of CPP for evaluating the negatively reinforcing (i.e. analgesic) properties of drugs. Initially, arthritic rats showed an enhanced choice preference to a morphine paired chamber relative to drug treated controls [39] and it was concluded that this was because morphine was more rewarding in the context of pain. However, this interpretation was somewhat obscured in that non-inflamed rats showed an equivalent chamber time preference, and only the choice measure was significant. Sufka [39] describes some possible reasons; one being that choice testing could be a more relevant measure of response strength, whereas time could be influenced by factors unrelated to reinforcement. The intrinsically rewarding properties of morphine also presented challenges of interpreting unconditioned responding (the animals’ subjective state), and why choice was superior to time. Then, however, a series of 6 discrete trials was used over 3 days of testing. Although we could not conduct similar choice trials due to a greater burden of conditioning and testing, we investigated the surrogate measure of entrances to each compartment over the first 60 s of each preference test. This, however, did not prove more informative than time preference so the results were not presented. Sufka’s original conclusion regarding the value of the CPP methodology in the context of negative reinforcement was ultimately strengthened when a Bradykinin B_1_ antagonist was used, which unlike morphine, had no intrinsically rewarding effects but was also preferred by formalin treated versus control rats [40]. This highlighted CPP as a method that could be applied outside of its usual domain for testing the abuse liability of drugs, and it has since been applied in several neuropathic and inflammatory pain models, all of which have successfully used ‘time’ as the dependent measure of unconditioned responding [41]–[43], [45], [46]. These studies, and the results presented here show CPP testing is one of the very few methods with potential for effectively differentiating between the sensory (nociceptive) and affective dimensions of pain. We know of only one previous study where the CPP methodology has been used to assess cancer pain [58], and this failed to show CPP to morphine 24 days after development intra-plantar melanoma. Notably, they used a much higher morphine dose than in our study (10 mg/kg), so the lack of effects may have been due to tolerance issues or drug-induced hyperalgesia; both very common effects of repetitive opioid treatment. There was little evidence for either in our study since there were no differential behavioural responses to terminal morphine treatment in pre-exposed or drug naïve mice. Additionally, the morphine preference of controls changed relatively little from 10 to 4 days before euthanasia, whereas drug-seeking significantly increased over the same time period in the tumour mice. Tolerance would presumably have increased drug seeking in controls, and conversely, hyperalgesia should have prevented this in cancer mice. In reality, however, we may actually have underestimated when pain began because the first time that the cancer-bearing morphine group showed significantly elevated morphine seeking was 7 days (3 preference tests) before euthanasia. As learning this association could conceivably have required several conditioning cycles, the actual time when pain (and its relief) became salient cues may have been at some earlier time point.

Since the tumour model described by Betourne et al. [58] should still have caused localised pain, another possible explanation of their negative findings could be that the mice were so incapacitated that they were insufficiently motivated make a chamber choice. However, other design differences could also offer an explanation. As in Sufka’s original study [39], all other studies where the CPP paradigm has been applied to pain have used the traditional approach of conditioning (using one or several drug exposures) followed by preference testing. We applied CPP testing where there could be severe, but slowly developing pain, but additionally, we wished not only to assess its presence but capture its onset. This meant applying a radical design change, incorporating repeated sets of conditioning trials interspersed with multiple preference tests (see Figure 1). So far as we are aware this approach has not been used previously and our positive result may, at least in part, have been due to the more frequent conditioning trials consolidating the association between pain and drug treatment. Our method of processing results by matching mice according to the total number of conditioning and test cycles undertaken, though again unconventional, may also have helped clarify the main effects. As in the behaviour study, this was needed to minimise the impact of the variable tumour growth. This variation meant mice were enrolled in the study at different times, with the net consequence that we could not be certain that animals entered the study at equivalent stages of cancer development. Using the data matching procedure we only examined CPP scores during the likely critical study time of the 10 days before euthanasia. Although this method of processing the data was not ideal, it does not detract from our main conclusion that the increased preference for morphine in cancer mice relative to controls indicated the emergence of pain.

Another study objective was to investigate the need for refinements if there was evidence of pain following tumour implantation. To this end we tested if a pre-procedural dose of meloxicam (5 mg/kg) was an appropriate method of controlling post-surgical in the Hargreaves study. Mice seem to require relatively high doses of NSAIDs [26], [59] compared to the recommended rates for pain prevention in rats [23]. This was therefore a comparatively low pre-procedural dose, and was based on investigators’ concerns over possible later impacts on tumour development. Although a relatively common reason for excluding analgesics from cancer studies [60] there was no such effect here. In agreement with behavioural and other physiological evidence [23], [26] 5 mg/kg meloxicam also did not prevent post-surgical hyperalgesia, so switching to an alternative (e.g. buprenorphine) could be a refinement option, and is one that has already been shown to be less likely to interact with cancer study results [33] than other NSAIDs or opioids.

The effects of morphine were also assessed on euthanasia days when tumours were at an advanced stage. This delayed euthanasia by 75 minutes, but was justifiable since any positive effects could establish if the conditioning dose of morphine was analgesic. If so, this would provide further evidence of pain and establish if this was a refinement option in studies with a strong case for prolonging data collection. Although tumour-bearing mice travelled less and groomed more before morphine, our ability to detect any positive effects was confounded by several non-specific effects of drug treatment. Despite the pre-treatment differences, morphine increased the distance travelled and the frequency of bouts of inactivity and rearing in all mice including the controls (Figure 9). Such non-specific effects of opioids on behaviour are well documented [1], [61]–[64], and we continue to find these can subvert attempts to confirm beneficial effects in studies of post-operative pain in rats in mice [31], [65], [66]. However, morphine had little influence on grooming in normal mice, and there were indications of post-treatment reductions in grooming in the cancer groups (Figure 9c). Grooming behaviour has previously been concluded to be important for assessing effects due to abdominal surgery or other manipulation likely to induce acute visceral discomfort [30], [31], [56], [67], cancer [68], arthritis [69] and also shows pattern alterations during stress [70]. Perhaps consistent with stress, the cancer-bearing mice were less frequently inactive (unable to rest) prior to morphine (Figure 9d), but again, post-treatment effects were similar in all mice so no positive outcomes were detectable.

To maximise the welfare of mice used in cancer studies the refinements resulting from this work would ideally be widely applicable; such as to soft tissue cancers in general. Present results support this, at least for bladder cancer, since we implanted cells via 2 very different approaches (one surgical, one not) yet still found a commonality in pain symptoms over the final 7–10 study days. An obvious general recommendation would be to monitor animals as closely as possible during this time, possibly adding hyperalgesia and/or behaviour assessments to standard end-point determinations. We cannot state the extent to which behaviour or nociceptive changes should necessitate euthanasia, but increased grooming and the highly significant effects on rearing during cancer development indicate both could be useful. Although the effects on rearing could be also have been caused by *malaise* or some associated lethargy, they still indicated compromised welfare. Nociceptive assessments, however, require specialised equipment (e.g. Hargreaves apparatus) and routine CPP testing would obviously be implausible. The simplest alternative is to rely on weight changes, and accordingly, current model-specific guidelines suggest animals with solid internalised tumours and showing ‘severe’ cachexia (amounting to between 15 to 20% of baseline body weight) should be euthanased [11]. However, all cancer-bearing mice presently remained within these limits, with the greatest weight loss in the behaviour study being in the tumour group (4.6%). In the CPP study we found the greatest loss was 5 g in a tumour bearing mouse during the final 10 study days. We predicted that the repeated morphine treatments might minimise differences between the tumour and control groups, but seemingly not. One possible explanation is that although both the control and tumour groups were palpated daily, those with tumours may have experienced additional discomfort. We have previously shown that palpation adversely affects the behaviour of rats with bladder cancer [71]. Whether this was currently the case is speculative, but it is nevertheless advisable to be cautious in the manner and frequency of palpation. Body weight assessments should also be adjusted for increased mass due to progression of cancer. Although this was attempted it was found to be difficult, as in any model where tumours are internalised. In any event there was no effective relationship between body weight changes, *ex-vivo* tumour burden and survival (measured by study days from detection to euthanasia). Thus, even if palpation was an effective measure of burden, this would not have been useful in forecasting the need for euthanasia. A more likely scenario was that pain severity and survival were more closely governed by the tissues impacted on during cancer growth rather than burden *per se*. A better approach would be to use more sophisticated methods such as imaging tumours. We plan to use this in future studies to investigate relationships between tumour morphology and welfare, and the general applicability of the CPP and other findings where tumours grow in tissues where they are differentially likely to cause pain. As the majority of evidence indicated the mice were painful despite only moderate weight loss, we suggest protocols relying on this as a primary welfare indicator might consider a more conservative end-point; such as *any* consistent weight loss over a 2 to 3 day period in an otherwise healthy mouse. In studies where more consistent tumour growth is achieved and study duration can be estimated more accurately, our results suggest end-points might be brought forward by at least one week. The precise time is obviously still difficult to identify, but experienced care staff can be exceptionally capable in estimating survival.

A fundamental 3Rs principle is to obtain maximum from the least number of animals, but without compromising scientific integrity. By applying several different assessment methods we hoped to detect cancer pain or other negative impacts on welfare as reliably as possible. Using separate study groups, although a possible weakness, had the major advantage of minimising the burden of testing on individual mice. Indeed, the impact of high intensity testing may be the reason that at least one report has emerged where a range of different ‘quality of life’ assessments failed to demonstrate negative consequences in a rat neuropathic pain model [50]. We believe the present series of experiments achieved a good level of precision due to the consistency of findings despite some substantial methodological variations. Overall, our studies were conducted in a manner that closely complied with the 3Rs principle of reduction; the majority of data being collected in mice already undergoing cancer trials. However, this meant studying the female mice our colleagues routinely use; due to the greater ease of implanting tumours via the urethra. Oestrus stage could therefore have impacted on our results depending on what stage coincided with conditioning, such as by altering the sensitivity of the mice to morphine. The mice were singly housed, but not isolated. They were also fully acclimated and never in contact with male mice, thus over the course of the study the mice would have undergone several oestrus cycles. It was not feasible to attempt to determine the impact of this on results due to the additional sampling that would have been required. We could have implemented measures to disrupt or halt oestrus, however, the relatively frequent morphine treatments may have caused this already. Determining oestrus stage would also have been very time consuming, and could also have been counterproductive to results. There is extensive literature on sex and strain differences in response to pain and to drug treatment [72]–[76], so ideally we would repeat the work to establish if the CPP findings generalise to male mice also. However, this would necessitate either surgical tumour implantation, or the more invasive approach of catheterising the urethra of males, both of which could render results questionable. Overall, we argue that it is unlikely that only using female mice significantly weakened our main findings. It could also have been advantageous had we been able to apply the mouse grimace scale (MGS) as an adjunct to the various other measurement parameters [59], [77], [78]. However, the work was completed prior to its development, and the quality of video recording in the behaviour study, together with the method of filming for analysis by HCS meant the data were not suitable for retrospective analysis using this approach.

## Conclusions

The essence of this work was to address a recently highlighted need for more appropriate (systems-based) methods of analgesic drug screening, and the development of more relevant translational pain models. We used a common mouse cancer model and multiple test methods to simultaneously address a second but no less important aim. This was to establish if cancer has a negative impact on the welfare of mice (predictably caused by pain), as this would demand greater effort in refining present end-point guidelines. The work constitutes the most comprehensive series of studies so far undertaken to determine whether bladder cancer is painful to mice. We showed reduced body condition and weight, changes in spontaneous behaviour, and the development of peripheral sensitivity (hyperalgesia) as tumours developed. Added to this we used the CPP paradigm as a means of offering mice the opportunity to self-report on any awareness of pain. Those with cancer spent longer in an environment where they previously experienced the effects of morphine than mice conditioned to morphine alone. Another critical finding was that morphine seeking was closely linked (increased) with tumour burden. However, the validity of our conclusion that these effects indicated pain hinges on whether mice sought morphine as a result of its association with pain relief. It also assumes that the various other changes provide complementary evidence of this. We argue that according to the triangulation principle these results collectively provide convincing evidence of the presence of clinical-type pain in this mouse cancer model. If not pain, there was at least very strong evidence of negative impacts on welfare highlighting a need for a range of possible refinements. Mice in this bladder cancer model could benefit from removal at least 7, and possibly even 10 days prior to the scheduled end-point. Relying only on weight changes or other model-specific signs may not be sufficient to ensure mice do not experience pain before they are euthanased. Rearing deficits may be a useful early indicator of the onset of adverse effects. We found no evidence that provision of morphine was detrimental either to cancer development or to the welfare of mice. In mice implanted surgically there were also no obvious effects of a pre-surgery dose of meloxicam either on post-surgery nociception or tumour development. A more refined surgical protocol might be one using a more appropriate dose of an NSAID for pain prevention, but this would require preliminary work to determine how these or other pharmacological candidates might impact on result validity. However, depending on the sensitivity of required outcomes, future studies of this type might involve buprenorphine; where it has been shown in at least one cancer study to have negligible or even beneficial effects in terms of both welfare refinement and result validity [33]. In light of the numerous other examples where CPP testing has now been applied to assess centralised pain in models other than cancer, we also conclude that CPP testing offers a method of increasing the translational relevance of the results of analgesic screening studies.
